# Effect of Power Line Interference on Microphone Calibration Measurements Made at or Near Harmonics of the Power Line Frequency

**DOI:** 10.6028/jres.112.008

**Published:** 2007-04-01

**Authors:** Randall P. Wagner, Victor Nedzelnitsky

**Affiliations:** National Institute of Standards and Technology, Gaithersburg, MD 20899-8220

**Keywords:** acoustic calibrations, acoustic measurements, calibration, electronic instrumentation, microphone calibration, microphone pressure calibration, microphone reciprocity calibration, power line frequency, power line interference, pressure reciprocity calibration of microphones

## Abstract

The electrical measurements required during the primary calibrations of laboratory standard microphones by the reciprocity method can be influenced by power line interference. Because of this influence, the protocols of international inter-laboratory key comparisons of microphone calibrations usually have not included measurements at power line frequencies. Such interference has been observed in microphone output voltage measurements made with a microphone pressure reciprocity calibration system under development at NIST. This system was configured for a particular type of standard microphone in such a way that measurements of relatively small signal levels, which are more susceptible to the effect of power line interference, were required. This effect was investigated by acquiring microphone output voltage measurement data with the power line frequency adjusted to move the frequency of the interference relative to the center frequency of the measurement system passband. These data showed that the effect of power line interference for this system configuration can be more than one percent at test frequencies harmonically related to the power line frequency. These data also showed that adjusting the power line frequency to separate the interference and test frequencies by as little as 1.0 Hz can reduce the effect of the interference by at least an order of magnitude. Adjustment of the power line frequency could enable accurate measurements at test frequencies that otherwise might be avoided.

## 1. Introduction

Electronic instrumentation powered by, or operated near the influence of, the alternating-current power grid is subject to interference at harmonics of the power line frequency (i.e., 50 Hz or 60 Hz), including the fundamental. Even though such instrumentation is designed to minimize the magnitude of this interference, it can be problematic, especially in systems comprising interconnected instruments. When measurements of small signal levels are made at or near these harmonic frequencies, and extremely good accuracy is desired, this interference can be a cause for concern. Electrical measurements required during the primary calibrations of laboratory standard microphones by the reciprocity method are an example. Because of this interference, the protocols of international inter-laboratory key comparisons of microphone calibrations usually have not included measurements at power line frequencies. In addition, suppositions regarding measurements at 250 Hz (the 5th harmonic of 50 Hz) have been expressed [1]. Power line interference has been observed in microphone output voltage measurements made with a microphone pressure reciprocity calibration system under development at NIST. To investigate the effect of this interference on these measurements, data were acquired with the system operated at several different power line frequencies to move the frequency of the interference relative to the center frequency of the measurement system passband. This center frequency was always set equal to the test signal frequency. These data were then used to quantify the effect of the power line interference on the accuracy of the measurements and to determine the adjustment in power line frequency required to mitigate this effect. The numerical values obtained apply only to this particular system when configured as described herein, but exhibit trends similar to those that would be expected for data from similar systems.

## 2. Reciprocity Microphone Calibrations

For a specified frequency of a sine-wave signal, the pressure response of a microphone is the ratio of its open-circuit output voltage to the sound pressure uniformly acting on the exterior part of the diaphragm. This response is often determined for primary standard condenser microphones using the method of pressure reciprocity calibration [2,3] specified in national [4] and international standards [5]. This method involves measurements on acoustically coupled pairs of microphones. Each pair includes a transmitter (microphone electrically driven to produce sound) and a receiver (microphone producing an output voltage in response to the sound pressure at its diaphragm), installed at opposed ports in a gas-filled test cavity bounded by the microphone diaphragms and the walls of a cylindrical coupler. The test cavity volume is a parameter included in the equations used to determine the microphone sensitivities. The uncertainties in the calibrated microphone sensitivities are, therefore, influenced by the uncertainty in the test cavity volume. This volume is determined by the coupler volume, the microphone front cavity volumes, and the equivalent volumes related to the acoustic impedances of the microphone diaphragms. Because a large part of the uncertainty in the test cavity volume is due to the uncertainties in these front cavity and equivalent volumes, their contribution to the uncertainty in the test cavity volume can be reduced by increasing the coupler volume.

As part of the calibration process, the receiver output voltage is measured. Influenced also by other variables, such as the barometric pressure and the temperature of the gas in the test cavity, receiver output voltage is approximately inversely proportional to the test cavity volume. Therefore, when larger couplers are used to reduce the uncertainty due to this volume, the receiver output voltage is decreased. Measurement of this decreased voltage is more susceptible to the effect of power line interference components in the noise floor.

## 3. Measurement System

**Fig. 1:**
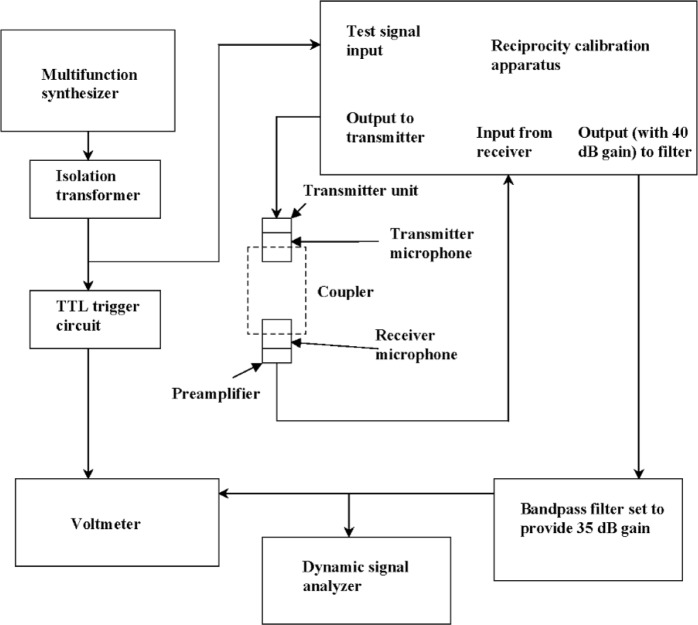
Block diagram of the microphone pressure reciprocity calibration system under development at NIST as it was configured to study the effect of power line interference on microphone output voltage measurements. The system configuration included a power source (not shown) capable of operating at frequencies between 45 Hz and 500 Hz inclusive, adjustable in 0.1 Hz increments. APC (not shown) was also included to control the multifunction synthesizer, reciprocity calibration apparatus, bandpass filter, and voltmeter over an IEEE-488 bus.

Figure 1 shows a block diagram of the microphone pressure reciprocity calibration system as it was configured to study the effect of power line interference on microphone output voltage measurements. The system included a Behlman P1352 AC Power Source^1^ that supplied power to all of the other system components. This power source is capable of operating at frequencies between 45 Hz and 500 Hz inclusive, adjustable in 0.1 Hz increments. A Hewlett Packard (HP) 8904A Multifunction Synthesizer supplied a 2 V sinusoidal test signal through an isolation transformer to a Bruel and Kjaer (B&K) Type 5998 Reciprocity Calibration Apparatus (RCA), which is part of the B&K Type 9699 Pressure Reciprocity Calibration System [6]. The RCA amplified the test signal by 6 dB and routed it to the transmitter. The RCA also provided the polarizing voltages required to operate the transmitter and receiver, and was set to amplify the receiver output by 40 dB. The RCA signal ground switch was set to the “FLOAT” position. The amplified receiver output was passed from the RCA through a bandpass filter comprising cascaded Frequency Devices 90PF H8EY and 90 PF L8EY programmable eight-pole, six-zero high-pass and low-pass elliptic filters. For all measurements, the filter was programmed to provide 35 dB gain at its center frequency.

**Fig. 2:**
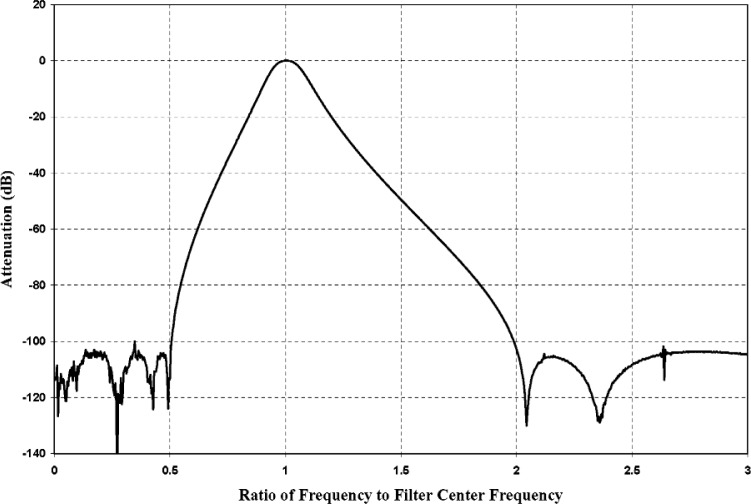
Frequency response of bandpass filter comprising cascaded Frequency Devices 90PF H8EY and 90PF L8EY programmable eight-pole, six-zero high-pass and low-pass elliptic filters.

The output of the filter was connected to an HP 3458A Multimeter, configured as a voltmeter in a mode that offers high accuracy for the measurement of periodic signals and requires a synchronous trigger. A trigger circuit that produced transistor transistor logic (TTL) pulses synchronized to the test signal was used to provide the voltmeter trigger. To center the filter passband on the test frequency, the corner frequencies of both the high-pass and low-pass filters were always set equal to the test frequency. A plot of the measured filter response in this configuration is shown in Fig. 2. This arrangement was used to measure the amplified and filtered receiver output voltage. The synthesizer, RCA, bandpass filter, and voltmeter were controlled over an IEEE-488 bus by a personal computer running a software package developed at NIST.

For spectral measurements of the system noise floor, an HP 3563A Dynamic Signal Analyzer was also connected to the filter output. These measurements were made by averaging 50 Fast Fourier Transform spectra. Each spectrum was characterized by 801 frequency lines from 0 Hz to 400 Hz (0.5 Hz line spacing), and was acquired using a time record length of 2.0 s and a Hann (also called a Hanning) window weighting function that resulted in an effective bandwidth of 0.75 Hz. For the analyzer measurements, the corner frequency of the high-pass filter was set to 20 Hz and the corner frequency of the low-pass filter was set to 400 Hz.

Both the transmitter and receiver were B&K 4180 microphones, which are Type LS2aP microphones that are specified in national [7] and international [8] standards. These microphones are less sensitive than the larger Type LS1P microphones [7,8] for which the relatively large coupler selected for this work was originally intended. This coupler has a nominal 15.0 mm length and a nominal 18.6 mm inner diameter. Adapters were used to install the smaller Type LS2aP microphones into the larger ports of the coupler.

This particular combination of microphones, adapters, and coupler was chosen to study the power line interference effect for a test cavity volume of about 5 cm^3^, which is about seven times larger than the test cavity volume that exists with the plane wave coupler of length 9.4 mm often used for low frequency calibration of Type LS2aP microphones. This increase in volume is large enough to significantly reduce the microphone calibration uncertainty component due to test cavity volume. The larger volume also reduced the receiver output voltage. Thus, the system was configured in such a way that measurements of relatively small signal levels, which are more susceptible to the effect of power line interference, were required.

Electrical connections to the two microphones were made through a B&K Transmitter Unit ZE 0796 and a B&K Receiver Preamplifier Type 2673T. The transmitter unit and receiver preamplifier were supplied with Modification WH 3405, which is the grounded shield configuration specified as the reference configuration in the relevant standards [4,5]. The transmitter unit, receiver preamplifier, microphones, adapters, and coupler were installed in a B&K Type UA 1412 Microphone Fixture with integral measurement chamber. This equipment helps to isolate the coupled microphones from acoustical noise during the measurements. During the course of the voltage measurements, barometric pressure and temperature were monitored. However, the observed changes in these environmental parameters were negligible, so no correction of the measured data was required.

## 4. Data Acquisition and Analysis

**Fig. 3:**
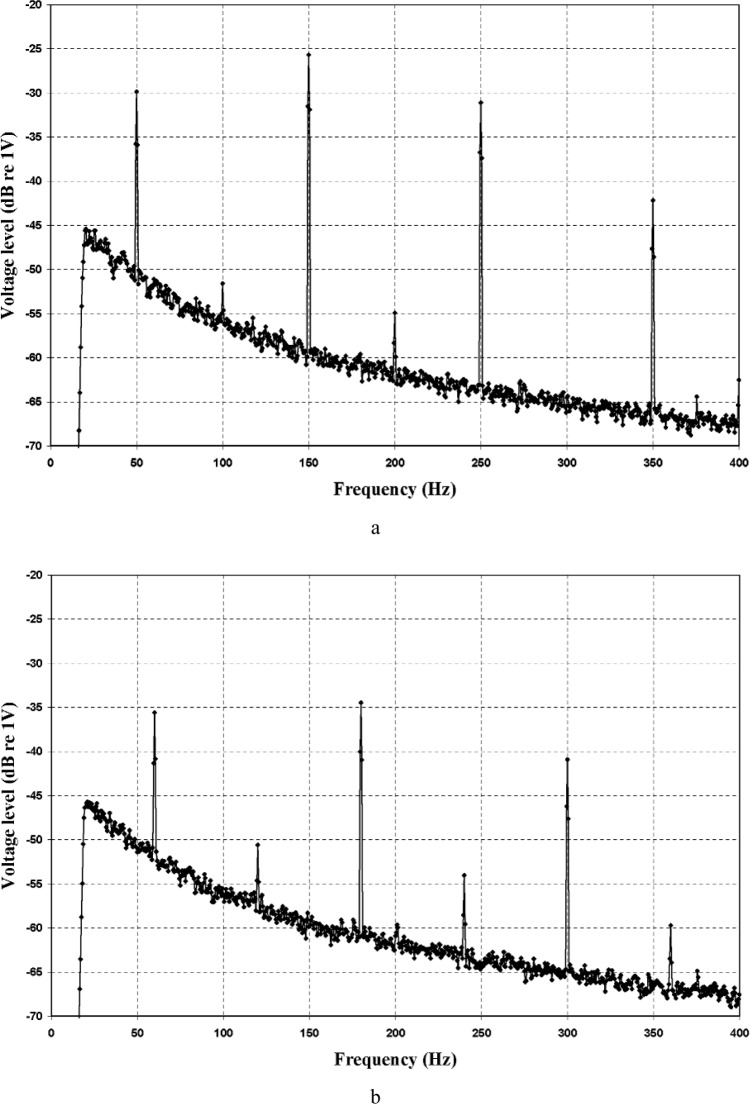
System noise floor spectra for (a) 50 Hz and (b) 60 Hz power line frequencies measured at the filter output with 75 dB total system gain and the microphone polarizing voltages turned on. Bandpass filter corner frequencies were 20 Hz and 400 Hz.

The system noise floor was measured in order to confirm the presence of power line interference and determine the relative magnitudes of the interference peaks. Spectra of the system noise floor for 50 Hz and 60 Hz power line frequency settings are shown in Fig. 3. The Appendix summarizes the uncertainty of these noise floor spectral measurement results. For these spectra, the microphone polarizing voltages were turned on. Both sets of data display baseline 1/f noise, and dominant interference peaks at the odd harmonics of the power line frequency. The appearance of these spectra is virtually identical to that of spectra acquired with the polarizing voltages turned off, indicating that the noise is primarily electrical rather than acoustical in origin.

For test frequencies of 63 Hz and 250 Hz, measurements of the amplified, filtered receiver microphone output voltage *V* (*f*) were repeated for various settings of the power line frequency *f*. Measurements at 63 Hz were done because this is the standard preferred frequency for acoustical measurements [9] that is nearest to 60 Hz, and was specified for a recent worldwide key comparison concerning pressure reciprocity calibration of Type LS2aP microphones [1]. This comparison also included measurements at 250 Hz, which is another standard preferred frequency for acoustical measurements. For each test frequency, $\bar{V}\left(f\right)$, the mean value of *V* (*f*) was calculated from twenty individual voltmeter readings acquired for a given power line frequency *f*. For all of these measurements, this value was approximately 1 V.

To quantify the power line interference effect for a given test frequency, we computed an error parameter *E*, in percent, given by

$$E=100\left(\frac{\sigma }{\bar{V}\left(f\right)}+\left|\frac{\bar{V}\left(f\right)-\bar{V}\left(f_{0}\right)}{\bar{V}\left(f_{0}\right)}\right|\right)$$ (1)

where *σ* is the standard deviation of $\bar{V}\left(f\right)$, and *f*_0_ is a reference power line frequency. For each test frequency, the reference power line frequency was the standard power line frequency (50 Hz or 60 Hz) whose nearest harmonic to the test frequency was the more removed from the test frequency.

**Fig. 4:**
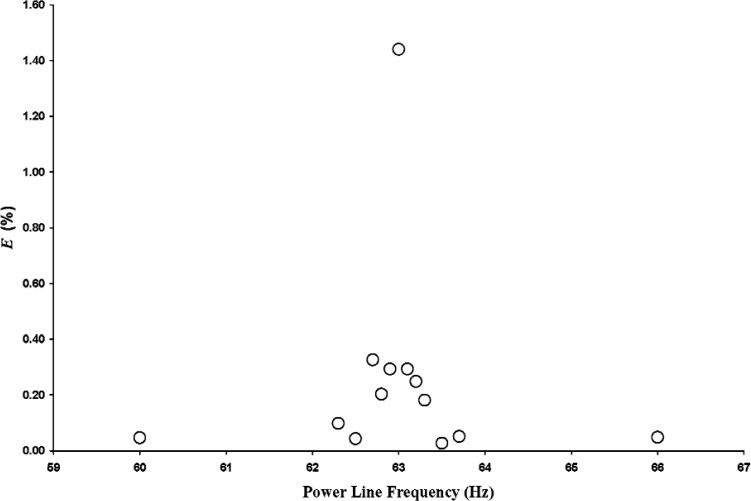
Error parameter *E* as a function of power line frequency for 63 Hz test frequency and for 50 Hz reference power line frequency.

For the 63 Hz test frequency, values of *E* calculated using 50 Hz as the reference power line frequency are shown in Fig. 4. The maximum value of *E* occurs at 63 Hz when the power line frequency and test frequency are equal. As the power line frequency is moved away from the test frequency in 0.1 Hz increments, *E* decreases dramatically. For power line frequencies removed from 63 Hz by at least 0.5 Hz, *E* is at least an order of magnitude less than its maximum value. At the standard power line frequency of 60 Hz, *E* decreases to about 3 % of the maximum value.

**Fig. 5:**
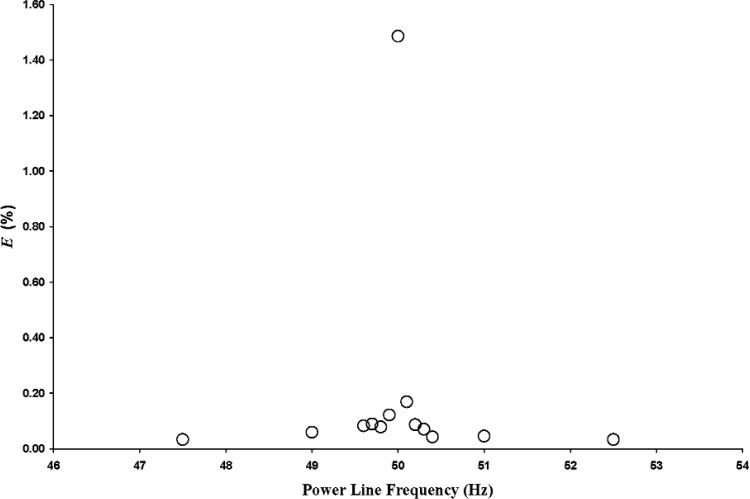
Error parameter *E* as a function of power line frequency for 250 Hz test frequency and for 60 Hz reference power line frequency.

For the 250 Hz test frequency, values of *E* calculated using 60 Hz as the reference power line frequency are shown in Fig. 5. As the power line frequency is moved away from the 50 Hz subharmonic of the test frequency, *E* decreases dramatically. For power line frequencies removed from 50 Hz by at least 0.2 Hz, *E* is at least an order of magnitude less than its maximum value. Because the interference peak at 250 Hz is the 5th harmonic of the 50 Hz power line frequency, a 0.2 Hz change in power line frequency results in a 1.0 Hz change in the interference frequency.

## 5. Conclusions

The effect of power line interference on microphone output voltage measurements made with a microphone pressure reciprocity calibration system under development at NIST was investigated by adjusting the power line frequency to move the interference frequency relative to the center frequency of the measurement system passband. The system configuration included a test cavity volume that is larger than usual to reduce the calibration uncertainty component due to this parameter. However, the larger volume results in a lower microphone output voltage because this voltage decreases with the increase in volume. In this sense, the system was configured in such a way that measurements of relatively small signal levels, which are more susceptible to the effect of power line interference, were required. System noise floor spectral data confirmed the presence of interference peaks at frequencies harmonically related to the power line frequency.

Measurements of *V* (*f*), the amplified, filtered receiver microphone output voltage, were made with power line frequency harmonics equal to, and removed from, the test frequencies. An error parameter, *E*, was defined [see Eq. (1)] to quantify the effect of power line interference on these measurements. Data for the 63 Hz test frequency revealed that, for power line frequencies removed from 63 Hz by at least 0.5 Hz, *E* is at least an order of magnitude less than its maximum value, which is at 63 Hz. At the 60 Hz standard power line frequency, *E* decreases to about 3 % of the maximum value. Data for the 250 Hz test frequency showed that at least an order of magnitude reduction in *E* occurred when the 5th harmonic of the power line frequency was removed from the 250 Hz test frequency by at least 1.0 Hz.

Though demonstrated for a highly specific application, adjustment of instrument performance by positive control of the power line frequency could be exploited with any instrumentation capable of operating over a range of power line frequencies. Because the vast majority of electronic instrumentation now in service is not restricted to a single power line frequency, this technique could be widely applied.

## Figures and Tables

**Table 1:** Summary of uncertainties in the voltage level data shown in the system noise floor spectra

Source of uncertainty	Relative standard uncertainty (%)
Repeatability (Type A)	6.8
Analyzer amplitude accuracy specifications (Type B)	4.9
Hanning window (Type B)	10.7

Combined relative standard uncertainty: 13.6 %	
Expanded (k=2) uncertainty: 27.2 %	
Expanded (k=2) uncertainty: 2.1 dB	

## Footnotes

1 Certain commercial equipment, instruments, or materials are identified in this paper to foster understanding. Such identification does not imply recommendation or endorsement by the National Institute of Standards and Technology, nor does it imply that the materials or equipment identified are necessarily the best available for the purpose.

## 6. Appendix – Uncertainty of Noise Floor Spectral Measurement Results

Expanded uncertainties in the voltage level data shown in the system noise floor spectra were developed by applying standard guidelines for evaluating and expressing uncertainties [10]. All of the components of standard uncertainty are summarized in Table 1 for these data. A Type A standard uncertainty, equal to the standard deviation of the mean level measured, was calculated to indicate the repeatability of the results. In addition, a Type B standard uncertainty was derived from the absolute amplitude accuracy of the analyzer by assuming a symmetric rectangular probability distribution, and using the applicable instrument specifications to estimate the upper and lower bounds of this distribution. Given this assumption, the resulting standard uncertainty is equal to one-half the width of the distribution multiplied by a factor of 0.58. A second Type B standard uncertainty was included to account for the fact that the Hanning window function was used. This uncertainty was also calculated by assuming a symmetric rectangular probability distribution, and using the applicable instrument specifications to estimate the upper and lower bounds of this distribution.
